# Asxl1 regulates optic cup development through interaction with Lhx2 and epigenetic modulation of Wnt signaling

**DOI:** 10.1080/19768354.2025.2542176

**Published:** 2025-08-04

**Authors:** Seungtae Moon, Nackhyuong Kim, A-Reum Kim, Kyeong Hwan Moon, Jin Woo Kim, Soo-Jong Um

**Affiliations:** a Department of Integrative Bioscience and Biotechnology, Institute of Bioscience, Sejong University, Seoul, Republic of Korea; b Department of Biological Sciences and KAIST Stem Cell Center, Korea Advanced Institute of Science and Technology, Daejeon, Republic of Korea

**Keywords:** Additional sex combs-like 1 (Asxl1), knockout, optic cup, Lhx2, Wnt signaling

## Abstract

The *additional sex combs-like 1* (*Asxl1*) gene is a chromatin regulator involved in transcriptional activation and repression. While Asxl1 plays a crucial role in various organ development, its role in ocular development remains unclear. Here, we analyzed *Asxl1* knockout (KO) mice and observed disrupted optic cup formation at embryonic day 10.5 (E10.5). RNA-seq of the E10.5 optic cup revealed dysregulation of Wnt signaling and early eye development genes. In further investigation using isolated cell from E10.5 retinal region, neuroepithelial stem cells from *Asxl1* KO embryos exhibited impaired proliferation and spheroid formation. To elucidate the transcriptional mechanism by Asxl1 in optic cup formation, biochemical assays demonstrated that Asxl1 binds the LIM domain of Lhx2, facilitating repression of Wnt1, Wnt2, and Wnt8b. Following ChIP analysis showed that the gain of function of Asxl1 increased repressive histone marks (H3K27me3, H3K9me3) and reduced active marks (H3K4me3) at Lhx2-binding motifs within the cis-regulatory regions of canonical Wnt ligand genes. These findings establish Asxl1 as a key epigenetic regulator of optic cup development by modulating Lhx2-mediated Wnt signaling, providing insights into congenital eye disorders.

## Introduction

Eye development is a complex and highly orchestrated process resulting in the formation of the vertebrate eye's intricate structure. In mice, eye development progresses through four distinct morphological stages: the optic pit (OP, E8.5), optic vesicle (OV, E9.5-E10), optic cup (OC, E10.5-E11.5), and lens formation (E11.5-E12.5) (Freund et al. 1996). These stages are regulated by a network of eye field transcription factors (EFTFs), which direct gene expression essential for the specification of retinal region. Early-stage EFTFs, including Pax6, Six3, Lhx2, Optx2, Hes1, and Otx2, modulate signaling pathways such as FGF, Wnt, and BMP to establish the retinal tissue (Adler and Canto-Soler 2007). Subsequently, axis determination of the OV involves coordinated regulation by Pax2, Pax6, Lhx2, Otx2, Mitf, and Vax (Schwarz et al. 2000; Chow and Lang 2001; Martinez-Morales et al. 2001; Horsford et al. 2005; Bharti et al. 2006; Canto-Soler and Adler 2006), while invagination of the OC is governed by Pax6, Hes1, andLhx2 (Tomita et al. 1996; Porter et al. 1997; Chow and Lang 2001; Lee et al. 2005; Canto-Soler and Adler 2006). Despite significant progress in identifying key transcription factors, the precise epigenetic mechanisms underlying their activity remain poorly understood.

LIM homeobox 2 (Lhx2) is a transcription factor with critical roles in eye development, as well as cerebral cortex development, lymphocyte differentiation, and hair follicle niche organization (Porter et al. 1997; Folgueras et al. 2013). Structurally, Lhx2 contains two LIM domains that mediate protein–protein interactions (PPIs) and a homeobox domain for DNA binding (Wang et al. 2014a, 2014b). Transcriptional activation or repression by Lhx2 depends onco-regulators that bind to its LIM domains (Mardaryev et al. 2011). Functional studies using Lhx2 knockout (KO) embryos have demonstrated its pivotal role in the transition from OV to OC during eye development (Wang et al. 2014a, 2014b). Notably, Lhx2 facilitates OC morphogenesis independently of other EFTFs (Folgueras et al. 2013). However, the transcriptional and epigenetic regulatory mechanisms involving Lhx2 remain largely unexplored.

Additional sex combs-like 1 (Asxl1) is a transcriptional co-regulator and the mammalian homolog of the Drosophila gene additional sex combs (Milne et al. 1999). While ASXL1's role as a tumor suppressor is well-documented, with multiple mutations implicated in various myeloid malignancies (Carbuccia et al. 2009; Boultwood et al. 2010; Chou et al. 2010), its broader biological functions are less understood. Previous studies using Asxl1 KO mice have revealed developmental defects, including microcephaly, cleft palate, alveolar maturation defects, glomerular podocyte abnormalities, and anophthalmia (Abdel-Wahab et al. 2013; Moon et al. 2015; Moon et al. 2018). Although the roles of Asxl1 in alveolar and podocyte development have been investigated (Moon et al. 2015; Moon et al. 2018), its contribution to ocular development and the associated transcriptional mechanisms remain unexamined.

In this study, we aim to delineate the role of Asxl1 in eye development by investigating its transcriptional and epigenetic regulatory mechanisms, with a particular focus on its interactions with Lhx2 and its influence on Wnt signaling.

## Material and methods

### Mouse strains and breeding

Asxl1-deficient mice (Asxl1<tm1a(EUCOMM)Wtsi>, MGI ID: 2684063, EMMAID: 03996) were used as previously described (Moon et al. 2018). All animal experiments were conducted with ethical approval from the Institutional Animal Care and Use Committee (IACUC) of Sejong University (protocol code SJ-20190702E2).

### Cell lines and culture conditions

Neuro-2a (N2a) and HEK293 T cells were cultured in DMEM (LM001-05, Welgene, Korea) supplemented with 10% heat-inactivated fetal bovine serum (FBS; #16000-044, Gibco, USA) and 1% antibiotic-antimycotic (#15240-062, Gibco, USA) in a humidified 5% CO₂ atmosphere at 37°C. For plasmid transfection, cells were maintained in antibiotic-free DMEM with 10% FBS. Transfection was performed using polyethyleneimine (PEI; #23966, Polysciences, USA) after a 10-minute incubation with plasmid DNA.

### Preparation of OCT-embedded tissue sections

Mouse embryonic facial tissues were collected in ice-cold 1× phosphate-buffered saline (PBS; #21600-010, Gibco, USA) and fixed in 4% paraformaldehyde (PFA; #158127, Sigma, USA) overnight at 4°C. Fixed tissues were washed with PBS, cryoprotected in 15% and 30% sucrose solutions (SB0498, Biobasic, Canada) at 4°C, and embedded in OCT compound (3801480, Leica, USA). Sections (6–10 μm thickness) were prepared for histological analysis. Hematoxylin and eosin (H&E) staining and immunofluorescence staining were performed using standard protocols.

### Isolation of neuroepithelial (NE) progenitor cells

Neuroepithelial progenitor cells were isolated as described previously (Gu et al. 2007). Briefly, optic vesicles (OV) were dissected from E9.5 embryos in HBSS (14025-092, Gibco, USA). Tissue was dissociated into single-cell suspensions by gentle trituration. Cells were centrifuged and resuspended in neural stem cell medium consisting of DMEM/F12 (D6421, Sigma, USA), 0.25% glucose (G8644, Sigma, USA), 1× B27 supplement (17504-044, Gibco, USA), 2 μg/ml heparin (H3393, Sigma, USA), 1× N2 supplement (17502-048, Gibco, USA), and 10 ng/ml human recombinant FGF2 (PHG0261, Gibco, USA). Cells (2 × 10⁴) were cultured in 60 mm petri dishes for 3–10 days at 37°C, adjusted based on empirically established spheroid size (100–150 μm in diameter).

### DNA constructs

Plasmids were constructed using standard molecular cloning techniques and verified by sequencing. 2×Flag- and Myc-tagged constructs were cloned into the pcDNA3 vector. GFP-tagged mutant constructs of mAsxl1 were generated in pEGFP-C3. For bacterial expression, GST- and His-fused proteins were cloned into pGEX4T-1 and pET-15b, respectively. pG4M-poly II and GAL4-TK-luciferase vectors were used for GAL4-DBD assays, while pGL2 was used for luciferase assays of Wnt1, Wnt2, and Wnt8b.

### Western blotting (WB) and immunoprecipitation (IP)

WB and IP assays were conducted as described previously (Moon et al. 2018). For WB, proteins from cell lysates were separated by SDS-PAGE, transferred to PVDF membranes (IPVH00010, Millipore, USA), and probed with the following antibodies: anti-Flag (M2, Sigma, USA), anti-Asxl1 (GTX127284, GeneTex, USA), anti-Lhx2 (GTX129241, GeneTex, USA), anti-β-actin (SC-47778, Santa Cruz Biotechnology, USA), and anti-GFP (M048-3, MBL, Japan). For IP, HEK293 cell lysates (1–2 mg) were incubated with antibodies (e.g. anti-Asxl1, anti-Flag, or anti-GFP) or mouse IgG (GTX213111-01, GeneTex, USA), followed by incubation with protein A/G-agarose beads (SC-2003, Santa Cruz Biotechnology, USA). Immunocomplexes were analyzed by WB and visualized using ECL reagent (16028, iNtRON Biotechnology, Korea) and an optical imaging system (Fusion Solo 4M, Vilber, France).

### β-Galactosidase (β-gal) Staining

Embryonic facial tissues were fixed in buffer containing 1% formaldehyde (F8775, Sigma, USA), 0.2% glutaraldehyde (G6257, Sigma, USA), and other components, followed by incubation in β-gal staining solution. Images were captured using a Leica microscope (DM IL system, Leica, Germany).

### RNA extraction and RT-qPCR

Total RNA was extracted from embryonic optic cup tissues or cells using Trizol reagent (10296028, Invitrogen, USA) and reverse-transcribed with M-MLV Reverse Transcriptase (28025013, Invitrogen, USA). Quantitative PCR was performed using a CFX96 real-time PCR detection system (Bio-Rad, USA), SYBR Green PCR Master Mix (QPK-201, Toyobo, Japan), and primers listed in Supplementary Table 1. mRNA levels were normalized to GAPDH as an internal control.

### RNA-seq and data analysis

For RNA-seq, 15 pairs of wild type or KO optic cup tissues were pooled separately. RNA quality was confirmed using an Agilent 2100 Bioanalyzer (Agilent, USA). Sequencing was performed on an Illumina HiSeq 2000 platform (Illumina, USA). Differentially expressed genes (DEGs) with 1.5-fold cutoff were identified using ExDEGA software (E-biogen, Korea), with results summarized in Supplementary Table 2. Gene Ontology (GO) analysis for functional analysis of RNA-seq was performed. To be more specific, Shiny GO V0.76 (released on 2nd September, 2022) was used for GO analysis and the result are presented in Supplementary Table 3. For additional information, Gene Set Enrichment Analysis (GSEA) was conducted and presented in Supplementary Table 4. The GSE99818 dataset from the GEO database was used to compare altered mRNA expression regulated by Asxl1 and Lhx2. Genes identified through GO and GSEA analyses are listed in Tables 5 and 6.

### Glutathione S-transferase (GST) pull-down assay

GST-fused Asxl1 protein was expressed in E. coli and purified using glutathione-Sepharose beads (GE Healthcare). His-tagged Lhx2 protein was purified using Ni-NTA agarose beads (Thermo Fisher Scientific). Proteins were incubated, and bound complexes were analyzed by WB using anti-His antibody (MBL, D291-3).

### Luciferase assay

N2a cells were co-transfected with pGL2 luciferase vectors, pcDNA, and a CMV-β-galactosidase expression vector. Luciferase activity was measured using the Luciferase Assay System (E1500, Promega, USA) and normalized against β-galactosidase activity detected with ONPG (ND0382, Biobasic, Canada).

### Chromatin immunoprecipitation (ChIP)

ChIP assays were conducted as previously described (Moon et al. 2018). N2a cells transfected with Flag-mAsxl1 plasmids were cross-linked with formaldehyde, sonicated, and immunoprecipitated using antibodies against Lhx2, Flag, Ezh2 (Active Motif, 39901, USA), H3K27me3 (Millipore, 07-449, USA), or HP1a (Millipore, 05-689, USA). The immunoprecipitated DNA was amplified by qPCR using primers specific to Lhx2-binding motifs in the cis-regulatory regions of Wnt1, Wnt2, and Wnt8b (Supplementary Table 7).

### Statistical analysis

All data are presented as mean ± SD from at least three independent experiments. Statistical comparisons were performed using paired t-tests, with significance set at *P* < 0.05 (*), < 0.01(**), and < 0.001(***).

## Results

### Optic cup impairment in Asxl1 KO mouse embryo

**Figure 1.**
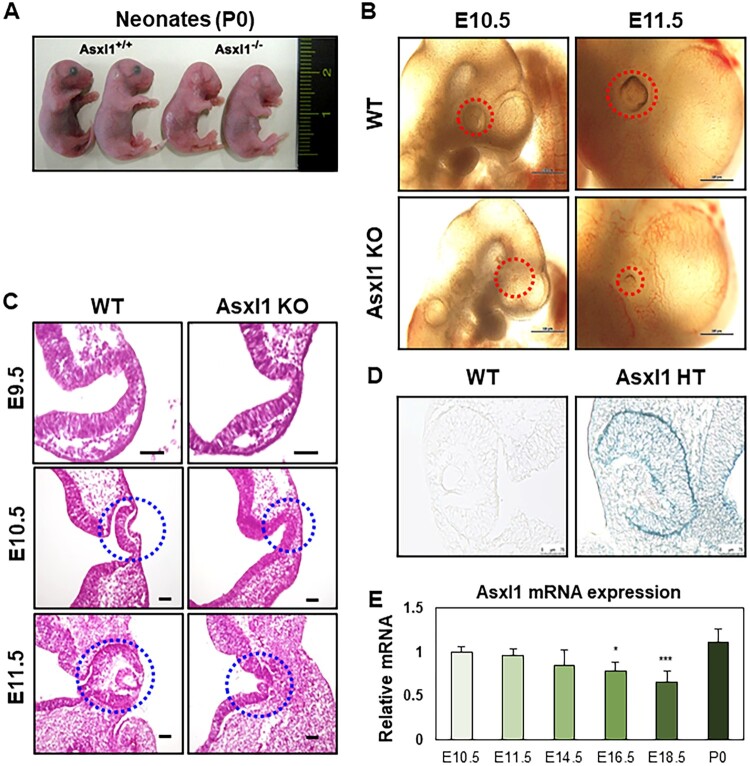
Histological and molecular analysis of eye development defects in Asxl1 KO mice. (A) Representative images of Asxl1 KO neonates, showing abnormal eye morphology. (B) Microscopic observation of the retinal region in Asxl1 KO embryos at E10.5 and E11.5. Red circles highlight the retinal region. (C) H&E staining of the optic cup and lens placode from E9.5 to E11.5 in Asxl1 KO embryos reveals impaired eye development. Scale bar indicates 100 um. (D) β-gal staining of the retinal tissue in Asxl1 KO embryos at E10.5 indicates disrupted structure formation. (E) RT-qPCR analysis of Asxl1 mRNA expression in the retinal region during embryonic development. Retinal tissues were dissected and processed for RNA extraction, cDNA synthesis, and RT-qPCR as described in the Materials and Methods section. Statistical analyses were performed as outlined in the Materials and Methods section (*n* = 3; *p < 0.05, ***p < 0.001).

Newborn Asxl1 KO mice were observed to lack eyes (Figure 1A). At E10.5, microscopic examination revealed the absence of a lens field in Asxl1 KO mice, while abnormal lens placode development was apparent by E11.5 (Figure 1B). To investigate these developmental defects, embryonic tissues were cryosectioned using OCT compound and analyzed histologically with hematoxylin and eosin (H&E) staining across various developmental stages (Figure 1C). At E9.5, the optic vesicle (OV) was visible in both wild-type (WT) and Asxl1 KO mice. However, by E10.5, while the optic cup (OC) had formed in WT mice, the OV persisted in Asxl1 KO mice. By E11.5, Asxl1 ablation ultimately resulted in a failure of lens formation.

To determine the expression pattern of Asxl1 during mouse embryonic eye development, β-galactosidase staining was used to confirm Asxl1 localization in optic cup at E10.5 (Figure 1D). Asxl1 expression was predominantly detected around the RPE, the leading edge of invagination during OC formation. To further analyze Asxl1 expression during eye development, RNA was extracted from eye tissue, and the relative expression level of Asxl1 was compared from E10.5 to P0 using RT-qPCR (Figure 1E). Since Asxl1 is a transcriptional coregulator, its loss likely caused OC malformation through alterations in the gene expression patterns of the retinal region.

### Aberrant gene expression patterns in Asxl1 KO mouse eye at E10.5

**Figure 2.**
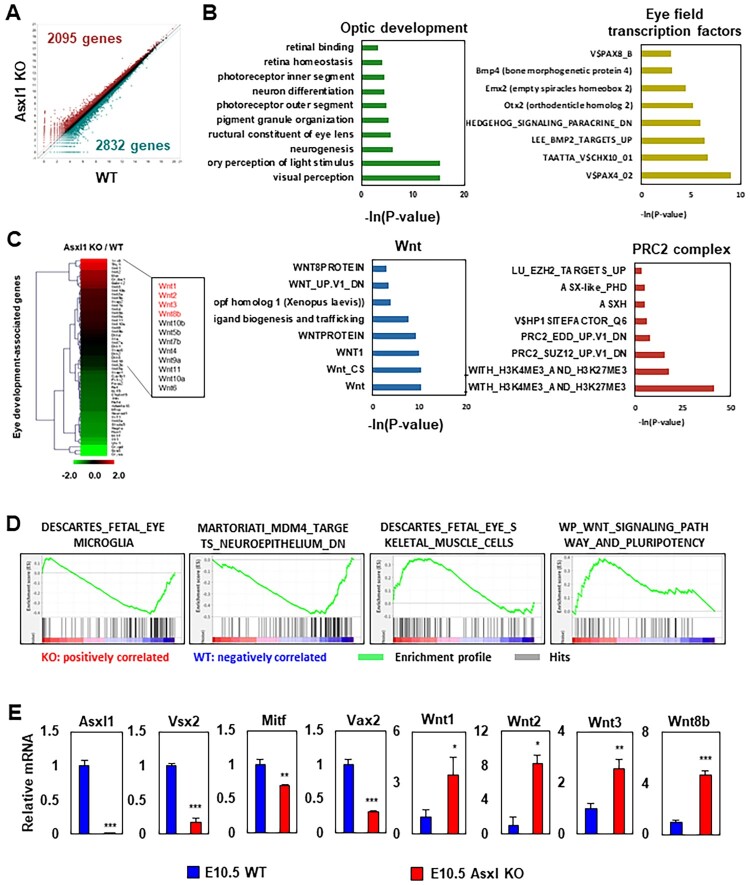
Gene expression analysis in the E10.5 optic cup of Asxl1 KO mice. RNA was extracted from the retinal tissue of WT and Asxl1 KO embryo, respectively. (A) Scatter plot showing differentially expressed genes (DEGs) in the E10.5 optic cup of Asxl1 KO mice. Relative gene expression was compared between WT and Asxl1 KO. Genes with a 1.5-fold change cutoff were considered altered. (B) Gene Ontology (GO) analysis of DEGs to explore the functional role of Asxl1 in the E10.5 optic cup. GO terms related to optic development, EFTFs, Wnt signaling, and the PRC2 complex were identified. The x-axis represents –ln(*P*-value) from the GO analysis. (C) Heat map showing the expression of eye development-related genes and Wnt ligands, in Asxl1 KO E10.5 optic cup. (D) GSEA of the Asxl1 KO E10.5 optic cup highlighting the enrichment of gene sets associated with retinal cell types (microglia, neuroepithelium, eye skeletal muscle) and Wnt pathway genes. (E) Validation of altered mRNA expression levels of Wnt ligands and other eye development-related genes in Asxl1 KO mice, normalized to Gapdh expression levels. Statistical analyses were performed as described in the Materials and Methods section (*n* = 3; *p < 0.05, **p < 0.01, ***p < 0.001).

To examine the transcriptomic changes associated with OC impairment due to Asxl1 ablation, RNA-seq analysis was performed on eye tissues from WT and Asxl1 KO mice at E10.5. Differential expression analysis identified 2,095 upregulated genes and 2,832 downregulated genes in Asxl1 KO mice (Figure 2A). Gene ontology (GO) analysis highlighted alterations in processes related to optic development, EFTFs, Wnt signaling, and PRC2 complex functions (Figure 2B). To gain further insight, genes frequently implicated in optic development were visualized using heatmaps (Figure 2C). Notably, several Wnt ligand genes were found to be upregulated in Asxl1 KO mice. GSEA revealed functional perturbations in eye-related cell populations (Figure 2D). Among the deregulated genes, Vsx2, Vax2, Mitf, Wnt1, Wnt2, Wnt3, and Wnt8b – all known to influence eye development – were identified and validated (Figure 2E). The canonical Wnt signaling pathway, which is crucial for eye development (Fujimura 2016), showed increased activation in Asxl1 KO mice. In particular, Wnt8b, a known suppressor of retinal progenitor cell (RPC) differentiation in early eye development (Liu 2012), was upregulated. These findings suggest that the OC impairment in Asxl1 KO mice may be attributed to elevated expression of canonical Wnt ligands.

RPCs arise from neuroepithelial progenitor cells (NPCs), with the canonical Wnt pathway influencing RPC self-renewal (Duparc et al. 2007; Fuhrmann 2008; Liu 2012; Fujimura 2016). NPCs were isolated at E9.5, and morphological differences in neurospheres were observed between WT and Asxl1 KO samples at passage three (Supplementary Figure 1A). Seven days after subculturing 2 × 10^4^ cells, Asxl1-ablated NPCs exhibited proliferative defects compared to WT NPCs (Supplementary Figure 1B) and failed to form neurospheres larger than 100 μm (Supplementary Figure 1C). Furthermore, the difference in mRNA expression between WT and Asxl1 KO NPCs was analyzed (Supplementary Figure 1D) and compared with the data shown in Figure 2E. Similar to the upregulation of Mitf in Asxl1-deleted eye tissues at E10.5, its expression was increased in cultured NPCs, whereas both Vsx2 and Vax2 showed opposite expression. In E10.5 Asxl1 KO eye tissue, Wnt1, Wnt2, Wnt3, and Wnt8b were upregulated. However, in E9.5 Asxl1 KO NPCs, only Wnt1 and Wnt8b were significantly upregulated, with moderate expression of Wnt2 and transcriptional repression of Wnt3.

To investigate whether Wnt pathway activation contributes to the impaired proliferation and spheroid formation observed in Asxl1 KO NEPs, we treated cells with WNT-C59, a canonical Wnt signaling inhibitor. WNT-C59 treatment had minimal effect on WT NEPs, but partially rescued the reduced proliferation in Asxl1 KO NEPs (Supplementary Figure 2A). Although the number of spheroids increased following WNT-C59 treatment, it remained lower than in WT NEPs (Supplementary Figure 2B and C). Interestingly, the size distribution of spheroids in Asxl1 KO NEPs was restored to WT levels (Supplementary Figure 2D). These results suggest that elevated Wnt signaling contributes, at least in part, to the NEP phenotypes caused by Asxl1 loss. However, the incomplete rescue implies additional pathways or cumulative epigenetic alterations may also be involved.

### Physical interaction between Asxl1 and Lhx2

**Figure 3.**
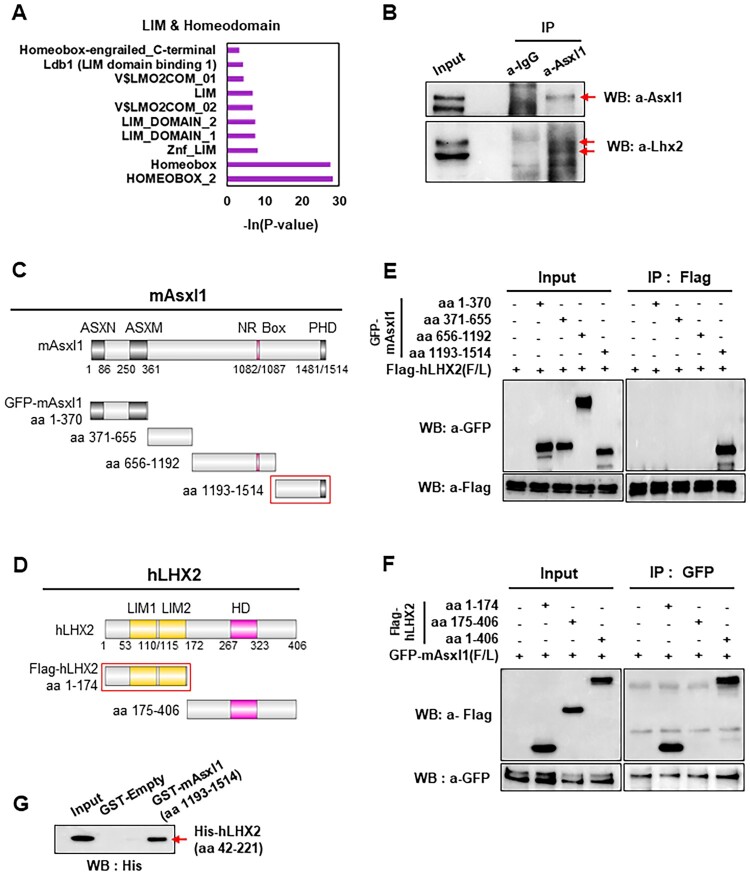
Protein-protein interaction between Asxl1 and Lhx2. (A) Gene Ontology (GO) analysis of differentially expressed genes related to LIM and homeodomains from E10.5 Asxl1 KO RNA-seq data. (B) Immunoprecipitation assay demonstrating the endogenous interaction between Asxl1 and Lhx2 in N2a neuroblastoma cells. (C, D) Schematic representation of the full-length and truncated forms of mAsxl1 (C) and hLHX2 (D) used in domain mapping experiments. (E, F) Immunoprecipitation assays for domain mapping of mAsxl1 and hLHX2, showing the specific regions involved in their interaction. (G) GST-pull down assay using bacterial-purified proteins: GST-empty, GST-mAsxl1 aa 1193–1514, and His-hLHX2 aa 42–221.

Asxl1, a nuclear transcriptional coregulator, has been hypothesized to interact with Lhx2 based on several findings: (1) Lhx2 KO mice phenocopied defective optic cup (OC) formation (Porter et al. 1997); (2) Lhx2 inhibits Wnt signaling, although the mechanism is unclear (Zhang et al. 2014); (3) a previous study identified an interaction between the LIM domain of Wtip and the C-terminus of Asxl1 (Moon et al. 2015); and (4) RNA-seq analysis of Asxl1 KO mice revealed differentially expressed genes related to LIM and homeodomains (Figure 3A). Immunoprecipitation (IP) in N2a cells confirmed the interaction between Asxl1 and Lhx2 (Figure 3B). Two bands were detected in the input using the anti-Asxl1 antibody; however, only the upper band was consistently immunoprecipitated. Based on our previous studies employing Asxl1 knockout-derived lysates and independent co-immunoprecipitation analyses (Moon et al. 2015; Moon et al. 2018; An et al. 2019), we confirm that the upper band corresponds to endogenous Asxl1, whereas the lower band likely represents a non-specific background signal. Mapping experiments using GFP-tagged subdomains of mAsxl1 and Flag-tagged hLhx2 revealed that the LIM domain (aa 1–174) of Lhx2 interacts with the C-terminus (aa 1193–1514) of Asxl1 (Figure 3C–E). Conversely, IP experiments with mAsxl1 fragments confirmed that the interaction occurred via the C-terminus of mAsxl1 and the LIM domain of hLhx2 (Figure 3F). A GST pull-down assay using purified GST-mAsxl1 (aa 1193–1514) and His-hLhx2 (aa 42–221) proteins (Supplementary Figure 3) further demonstrated that this interaction is direct (Figure 3G). Since both proteins regulate transcription, the Asxl1-Lhx2 complex is likely to play a role in eye development.

To confirm the functional association between Asxl1 and Lhx2 at the transcriptional level, RNA-seq data from E10.5 Asxl1 KO optic cup were compared with E14.0 Lhx2 cKO RPCs (dataset GSE99818). Despite differences in embryonic stage and tissue specificity, 815 genes were commonly upregulated (Supplementary Figure 4A), of which GO analysis revealed associations with Wnt signaling and PRC2 complex functions (Supplementary Figure 4B). GO analysis of 373 downregulated genes (Supplementary Figure 4C) indicated that the downregulated genes were linked to optic development and EFTFs (Supplementary Figure 4D). These observations suggest a functional and transcriptional cooperation between Asxl1 and Lhx2 in regulating the expression of target genes.

### Transcriptional repressive function of Asxl1-Lhx2 interaction

**Figure 4.**
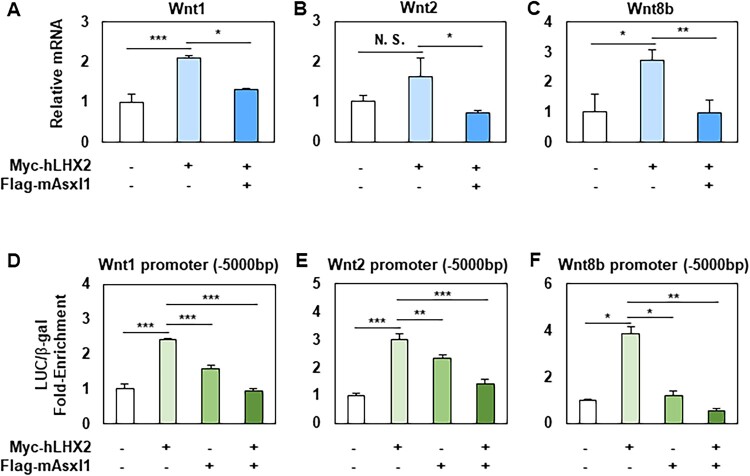
Transcriptional repressive function of Asxl1-Lhx2 interaction. (A-C) Regulation of mRNA expression levels of Wnt1 (A), Wnt2 (B), and Wnt8b (C) by Myc-hLHX2 and Flag-mAsxl1 in N2a cells. Data are normalized to Gapdh and presented as mean ± SEM (*n* = 3). (D-F) Luciferase assays using pGL2-Wnt1 −5000 bp (D), pGL2-Wnt2 −5000 bp (E), and pGL2-Wnt8 −5000 bp (F) luciferase reporters. CMV-β-gal construct was co-transfected for internal control. Data are presented as mean ± SEM (*n* = 3, *p < 0.05, **p < 0.01, ***p < 0.001, N.S. = not significant).

To explore the transcriptional regulation mediated by the direct interaction between Asxl1 and Lhx2, a fusion construct containing the GAL4 DNA-binding domain (GAL4-DBD) and the 1–174 amino acid region of human LHX2 (hLHX2) was generated. In a GAL4-DBD assay using 293 T cells, overexpression of GAL4-hLHX2 (aa 1–174) induced luciferase activity, but co-transfection with Flag-mAsxl1 significantly suppressed luciferase expression (Supplementary Figure 5). These results suggest that Lhx2 requires Asxl1 to function as a transcriptional repressor. To validate this mechanism in a cellular context, Flag-mAsxl1 and Myc-hLHX2 were overexpressed in N2a cells, and the mRNA levels of Wnt1, Wnt2, and Wnt8b were analyzed under different conditions (Figure 4A–C). Overexpression of hLHX2 alone increased Wnt ligand expression, whereas co-expression with Asxl1 repressed transcription of these Wnt genes. Further luciferase assays were performed using the −5000 bp cis-regulatory regions of Wnt1, Wnt2, and Wnt8b. Lhx2 alone significantly increased luciferase activity for all three cis-regulatory regions (Figure 4D–F). However, co-expression with Flag-mAsxl1 reduced enhancer activity, which confirms the Asxl1-Lhx2 complex as a negative regulator of Wnt transcription. To assess whether the Asxl1 aa 1193–1514 fragment affects Lhx2-mediated transcription, we performed luciferase reporter assays (Supplementary Figure 6). Co-expression of this fragment with Lhx2 resulted in transcriptional repression comparable to that of full-length Asxl1. These findings indicate that the Lhx2-binding region of Asxl1 is functionally sufficient to suppress Lhx2-driven transcription, rather than exerting a dominant-negative effect.

### Chromatin recruitment of Asxl1-Lhx2 to Wnt cis-regulatory regions

**Figure 5.**
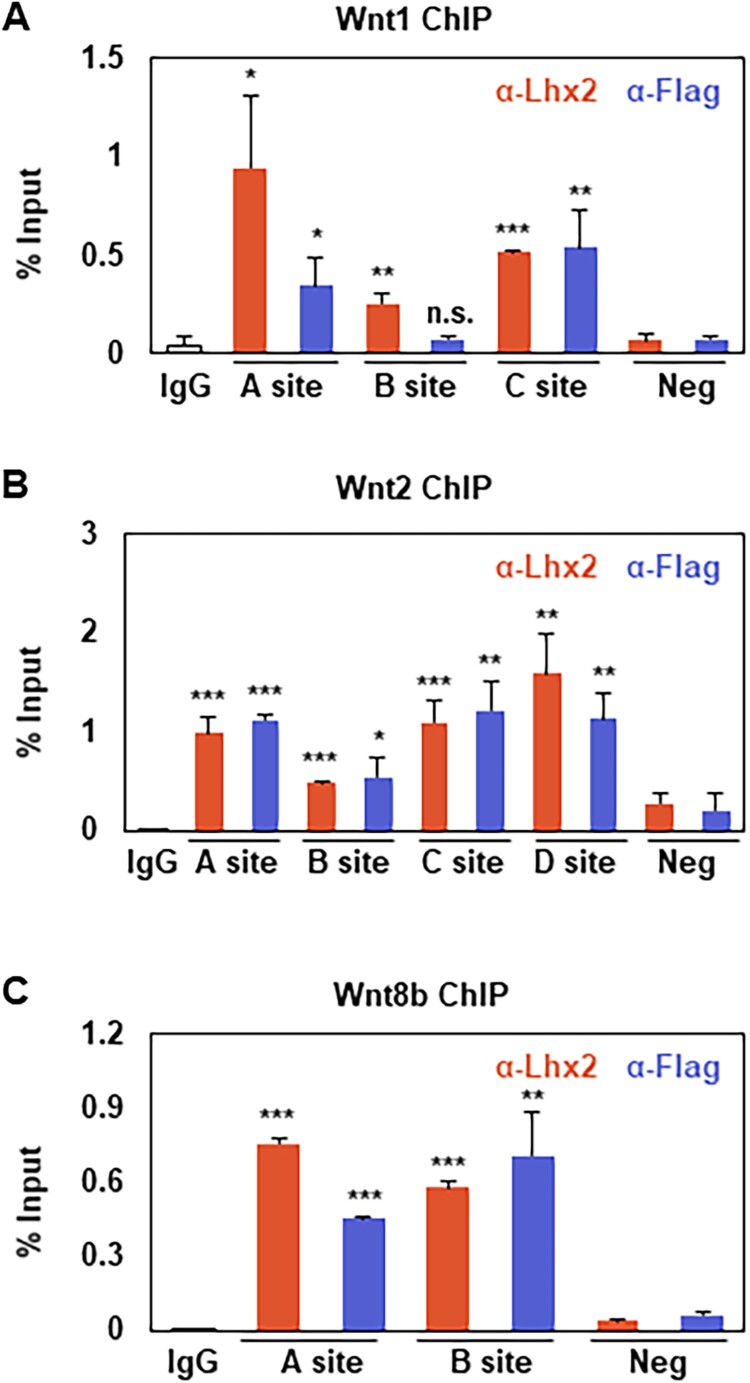
Chromatin interaction of Lhx2 and Flag-Asxl1 in N2a cells. (A–C) ChIP assay against Lhx2 or Flag in transfected N2a cells. Cells were transfected with Flag-mAsxl1 for 24 h prior to performing the ChIP assays targeting murine cis-regulatory regions of Wnt1 (A), Wnt2 (B), and Wnt8b (C). Primers were designed based on mm9 reference genome of UCSC and indicated in the schematic representation (Supplementary Figure 7). Statistics of ChIP-PCR were calculated as described in the material methods (*n* = 3) (*p < 0.05, **p < 0.01, ***p < 0.001, n.s. = not significant).

To investigate the transcriptional regulatory mechanism of Wnt1, Wnt2, and Wnt8b by the Asxl1-Lhx2 complex, primers were designed to target regulatory regions within 5000 bp upstream from each TSS were designed, focusing on the ‘TAATT’ DNA motif as a potential Lhx2 binding site (Supplementary Figure 7A–C) (Monahan et al. 2017; Muralidharan et al. 2017; Zibetti et al. 2019). N2a cells transfected with Flag-mAsxl1 were subjected to ChIP assays using antibodies against endogenous Lhx2 and Flag-mAsxl1 to identify protein–DNA interactions at these sites. Three TAATT sites (A, B, and C) were examined for the Wnt1 cis-regulatory regions. Both Lhx2 and Flag-mAsxl1 exhibited strong binding at sites A and C, whereas no Flag-mAsxl1 binding was detected at site B. Interestingly, Lhx2 binding at site B was weaker than at sites A and C, despite its proximity to site A, suggesting site-specific binding patterns within the cis-regulatory region (Figure 5A). In the upstream of Wnt2, which contains four TAATT sites, Lhx2 and Flag-mAsxl1 were found to bind at all sites. However, the binding intensity at site B was consistently weaker compared to the other sites (Figure 5B). The upstream of Wnt8b, which contains two TAATT sites, showed consistent binding of both Lhx2 and Flag-mAsxl1 at both sites with comparable intensity (Figure 5C). These results confirm the chromatin recruitment of the Asxl1-Lhx2 complex to the Wnt1, Wnt2, and Wnt8b regulatory regions, supporting its role in transcriptional regulation.

### Epigenetic regulation of Wnt1, 2, and 8b by Asxl1-Lhx2

**Figure 6.**
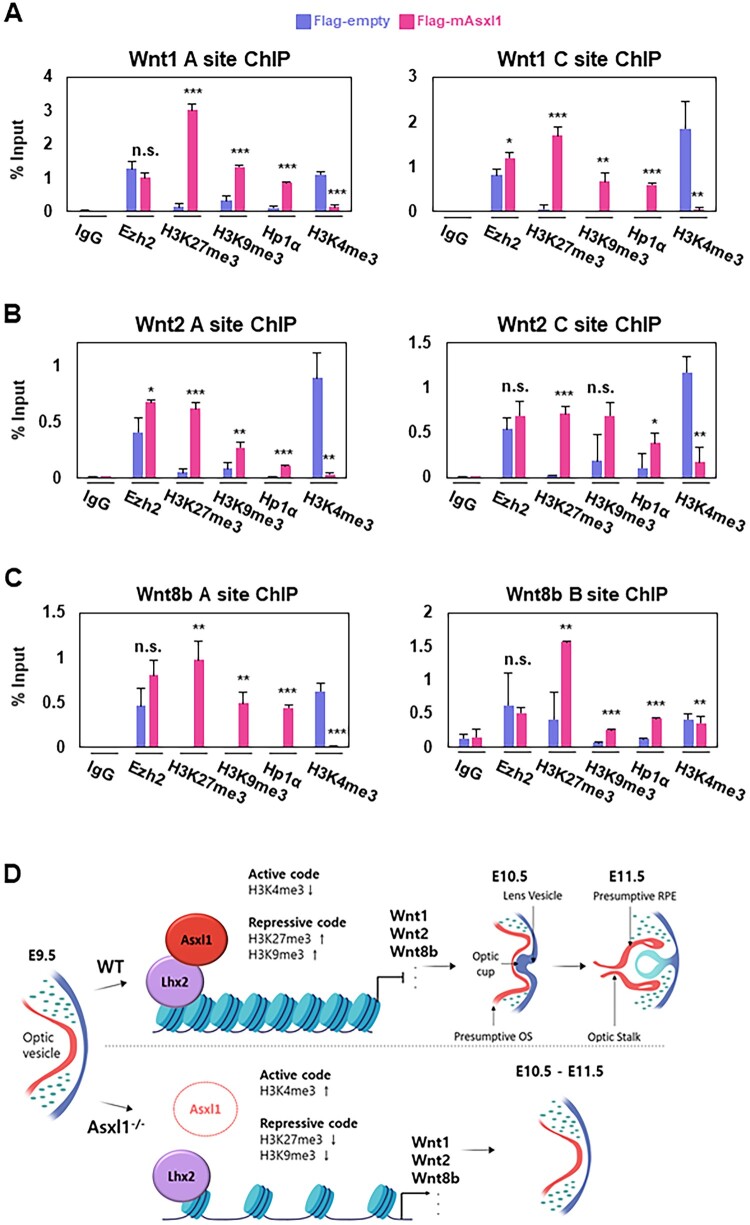
Epigenetic regulation of Wnt genes by Asxl1 in N2a cells. (A–C) ChIP-qPCR analysis of Ezh2, Hp1α, and histone modifications following Asxl1 overexpression in N2a cells. Sites A and C of Wnt1 (A) and Wnt2 (B), and site A and B of Wnt8a (C) were analyzed. Statistics were calculated as described in the material methods (*n* = 3) (*p < 0.05, **p < 0.01, ***p < 0.001, n.s. = not significant). (D) Graphical abstract summarizing the epigenetic role of Asxl1-Lhx2 during optic cup generation in eye development.

While Asxl1 and Lhx2 have been shown to bind to the regulatory regions of Wnt1, Wnt2, and Wnt8b, the exact epigenetic mechanisms underlying their transcriptional regulation remain undefined. Given the established role of H3K27me3, a histone modification catalyzed by the PRC2 complex, as a marker of Asxl1-mediated transcriptional repression (Sinclair et al. 1998; Wang et al. 2014a, 2014b; Moon et al. 2018; Patnaik et al. 2018; Rohatgi et al. 2018), its potential involvement in Wnt regulation was explored. Furthermore, the interplay between H3K27me3 and the active histone mark H3K4me3 (Lin et al. 2015; Kinkley et al. 2016; Liu et al. 2016; Taube et al. 2017; Iwagawa and Watanabe 2019), as well as the association of Hp1α with the repressive H3K9me3 mark and its known interaction with Asxl1, suggest that multiple chromatin modifications may contribute to this regulatory mechanism. To investigate how Asxl1 modulates chromatin states via Lhx2 at cis-regulatory regions of Wnt ligand genes, ChIP assays were performed to assess the enrichment of Ezh2 (a PRC2 component), H3K27me3, H3K9me3, Hp1α, and H3K4me3 (Figure 6A–C). In general, Asxl1 increased the accumulation of repressive histone marks (H3K27me3 and H3K9me3) and the heterochromatin factor Hp1α, whereas it decreased the enrichment of the active histone mark H3K4me3 at sites A and C of each cis-regulatory region of Wnt ligand genes. These experiments aim to elucidate the relationship between Asxl1-mediated chromatin modifications and the transcriptional regulation of Wnt1, Wnt2, and Wnt8b, providing insight into the epigenetic framework of Asxl1-Lhx2 complex activity.

## Discussion

In this study, we demonstrated that anophthalmia in Asxl1 knockout (KO) mice is caused by impaired optic cup (OC) formation at E10.5. The absence of the retinal pigment epithelium (RPE) layer and cornea in these mice highlights the critical role of invagination during eye development. The expression of Asxl1 in the early RPE region further suggests its involvement in regulating genes essential for ocular development. Despite this restricted expression, altered gene expression was observed across the broader retinal region, notably the upregulation of Wnt ligands, which are pivotal for neural progenitor cell (NPC) function and eye development (Liu 2012; Fujimura 2016). These findings suggest that the dysregulation of Wnt signaling pathways by Asxl1 ablation in NPCs may impair normal RPE development. Future studies focusing on other cell types involved in eye development will provide a more comprehensive understanding of these signaling pathways and their interconnections.

Comparison of our RNA-seq data with Lhx2 dataset GSE99818 strongly indicated that both Asxl1 and Lhx2 are associated with transcription of genes involved in eye development. We also uncovered a novel physical interaction between Asxl1 and Lhx2, with domain mapping showing that the N-terminus of Lhx2 (containing LIM domain) interacts with the C-terminus of Asxl1 (containing the PHD domain). This interaction suggests potential regulatory roles of Asxl1 with other LIM-containing proteins, such as Lhx2 or Wtip, broadening the functional scope of Asxl1. Using luciferase assays with the GAL4 system and cis-regulatory regions of Wnt ligand genes, we showed that while Lhx2 upregulates Wnt1, Wnt2, and Wnt8b expression, co-overexpression of Asxl1 and Lhx2 represses their transcriptional activity. These findings highlight the Asxl1-Lhx2 complex as a key transcriptional repressor, warranting further investigation into its activity in other cellular contexts and target genes.

At the level of epigenetic mechanism, Asxl1 and Lhx2 appears to modulate chromatin states at the cis-regulatory regions of Wnt ligand genes through histone modifications. We observed that H3K27me3, catalyzed by the PRC2 complex, increased at Lhx2 binding sites upon Asxl1 overexpression, suggesting a role for Asxl1 in enhancing Ezh2 enzymatic activity, rather than its recruitment. Additionally, H3K9me3 and Hp1α levels increased following Asxl1 overexpression, with Hp1α physically interacting with Asxl1. This interaction may stabilize H3K9me3 by preventing its removal by epigenetic erasers. Conversely, the active histone mark H3K4me3 decreased, reflecting the antagonistic relationship between H3K27me3 and H3K4me3. As summarized in Figure 6D, we conclude that Asxl1 coordinates with the transcription factor Lhx2 to epigenetically repress Wnt signaling during optic cup formation from the optic vesicle. These findings suggest that Asxl1 regulates chromatin dynamics through multiple histone modifications, though the precise mechanisms, particularly for H3K9me3 modulation, require further investigation.

Lhx2 and Asxl1 are co-expressed in the RPE and optic cup regions during early eye development, and knockout models of either gene show defects in optic cup formation (Hägglund et al. 2011). Although their expression persists beyond this stage, the most prominent phenotypes occur during optic cup formation, suggesting that the Asxl1–Lhx2 complex functions in a stage-specific chromatin or transcriptional context. To date, epigenetic cooperation between Asxl1 and Lhx2 has only been observed in the developing eye. Whether this complex operates in a constitutive or temporally restricted manner remains unclear. Lhx2 exhibits dynamic and spatially restricted expression during embryogenesis, including roles in the telencephalon and skin appendages (Rhee et al. 2006; Chou et al. 2009), whereas Asxl1 is broadly expressed from the embryonic stem cell stage and regulates multiple organ developmental processes (Moon et al. 2015; Moon et al. 2018; An et al. 2019). These partially overlapping expression profiles suggest that the Asxl1–Lhx2 interaction may represent a transient transcriptional module that forms in specific developmental contexts. The high conservation of Lhx2 between mouse and human (only four amino acid differences) also supports the functional relevance of this interaction across species. Our results imply that Asxl1 forms distinct regulatory complexes with different transcription factors in a tissue- and stage-dependent manner, with the Asxl1–Lhx2 complex being a specific example during optic cup development.

Despite these insights, the cellular functions of Asxl1 in optic cup development remain incompletely understood. Future studies should explore its role in retinal progenitor cell differentiation and glial cell arrangement for RPE formation. Advances in eye organoid technology may also allow detailed in vitro modeling of Asxl1 functions in retinal progenitor cells. At the molecular level, the physiological roles and broader epigenetic mechanisms of the Asxl1-Lhx2 interaction deserve further exploration. Taken together, this study provides valuable insights into the molecular mechanisms of Asxl1 in eye development and its potential involvement in the etiology of developmental eye disorders such as anophthalmia and microphthalmia. The findings presented here lay a foundation for future research and potential therapeutic strategies for these conditions.

## Supplementary Material

Supplementary table 5. Common target genes between E10.5 Asxl1 KO eye field and E14.0 Lhx2 cKO RPC.xlsx

Supplementary table 6. Gene list from GO analysis of common target genes between E10.5 Asxl1 KO eye field and E14.0 Lhx2 cKO RPC.xlsx

Supplementary Figures.docx

Supplementary table 7. Primer list for ChIP-qPCR.xlsx

Supplementary table 4. GSEA gene list from E10.5 Asxl1 KO eye field.xlsx

Supplementary table 1. Primer list for RT-qPCR.xlsx

Supplementary table 3. Gene list from GO analysis of E10.5 Asxl1 KO eye field.xlsx

Supplementary table 2. RNA-seq of E10.5 Asxl1 KO eye field 1.5 fold cut-off.xlsx
