# Supplementary material for: Asxl1 regulates optic cup development through interaction with Lhx2 and epigenetic modulation of Wnt signaling
Source: Anim Cells Syst (Seoul). 2025 Aug 4;29(1):488–501. doi: 10.1080/19768354.2025.2542176 (PMC12322997; doi:10.1080/19768354.2025.2542176)
Supplement: Supplementary Figures.docx [file TACS_A_2542176_SM4167.docx]

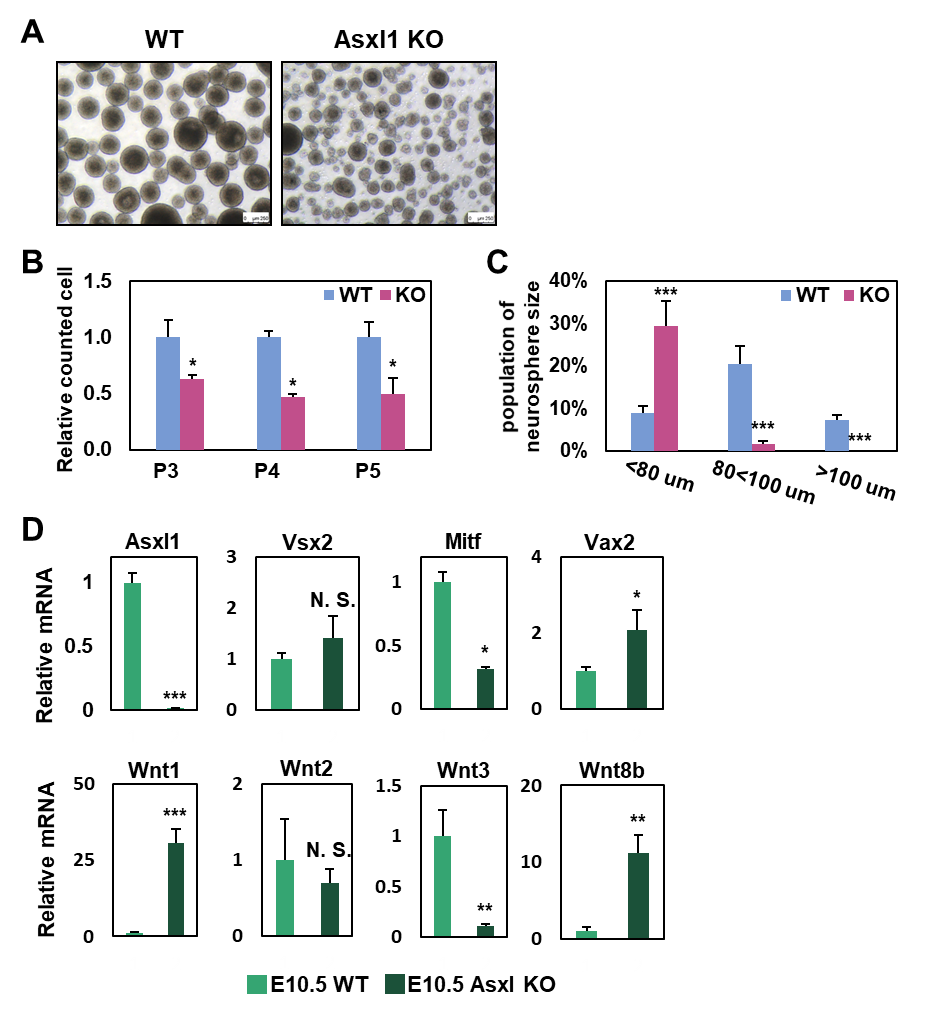


**Supplementary Figure 1. Defective neuroepithelial progenitor cells (NEPs) in Asxl1 KO embryos originating from the E10.5 eye field.** (A) Comparison of spheroid formation of **E10.5 NEPs** between **WT** and **Asxl1 KO.** (B) The cell number in **Asxl1 KO E10.5 NEPs** was significantly reduced when compared to **WT**. Cell counts were performed 7 days after culture. (C) Measurement of **E10.5 NEP spheroid size** in **Asxl1 KO**. Spheroid size was calculated from microscopic images taken after 7 days of culture. (D) Altered gene expression in **Asxl1 KO E10.5 NEPs** as revealed by RNA-seq. RNA was extracted from NEPs, cDNA was synthesized, and expression levels were normalized to **Gapdh**. Data (B, C, D) were calculated as described in material and method (n=3, *p < 0.05, **p < 0.01, ***p < 0.001, N. S. = not significant).


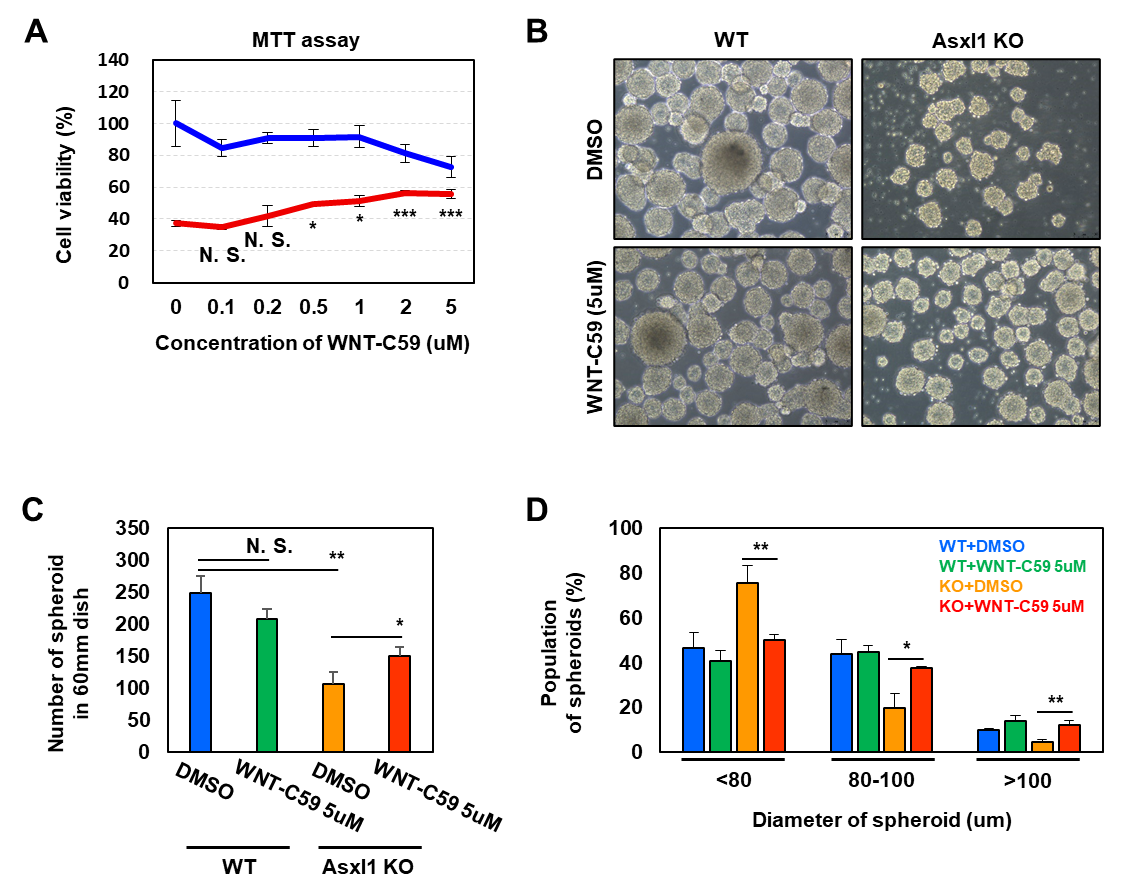


**Supplementary Figure 2. Partial rescue of proliferation and spheroid formation in Asxl1 KO NEPs by WNT-C59 treatment.** (A) MTT assay of WT and Asxl1 KO NEPs treated with or without WNT-C59 (HY-15659, MCE) for 72 h. Cells (4,000/well) were seeded in RV-96 plates. Cell viability was compared between WNT-C59-treated and untreated Asxl1 KO NEPs (n = 3). (B) Representative images of spheroid formation from E10.5 WT and Asxl1 KO NEPs 7 days after WNT-C59 treatment. (C) Quantification of spheroid numbers per dish (n = 3). (D) Distribution of spheroid sizes in each group (n = 3). Data are presented as mean ± SD. *p < 0.05, **p < 0.01, ***p < 0.001, N.S., not significant.


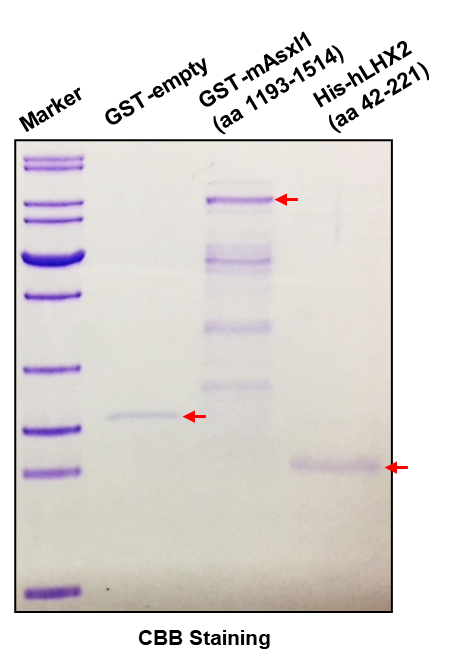


**Supplementary Figure 3. Coomassie Brilliant Blue (CBB) staining of purified proteins.** Purified proteins from **GST-empty**, **GST-mAsxl1 aa 1193**–**1514** and **His-hLHX2 aa 42**–**221** were stained with **CBB** after SDS-PAGE. **Red arrows** indicate the sizes of the purified target proteins.


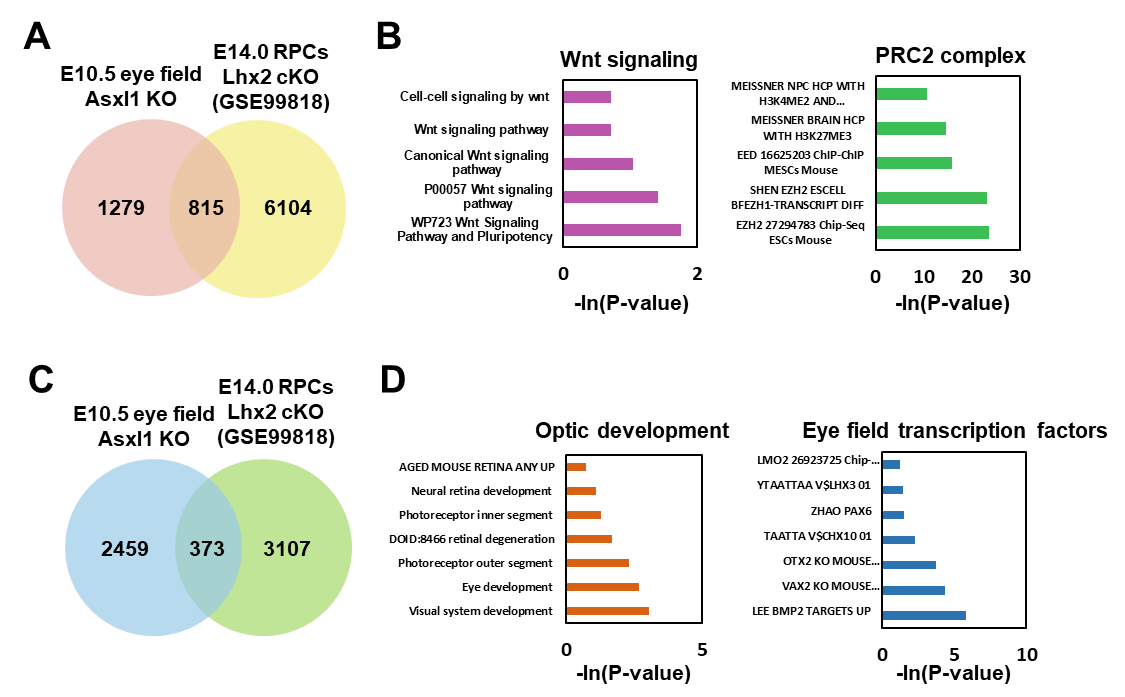


**Supplementary Figure 4. RNA-seq comparison between E10.5 Asxl1 KO eye field and E14.0 Lhx2 cKO RPCs.** (A) Commonly upregulated genes between **E10.5 Asxl1 KO eye field** and **E14.0 Lhx2 cKO RPCs**. (B) **GO** analysis of common upregulated genes. **GO** terms related to **Wnt signaling** and **PRC2 complex** were detected. (C) Common downregulated genes between **E10.5 Asxl1 KO eye field** and **E14.0 Lhx2 cKO RPCs**. (D) **GO** analysis of common downregulated genes. **GO** terms related to **optic development** and **EFTFs** were found.


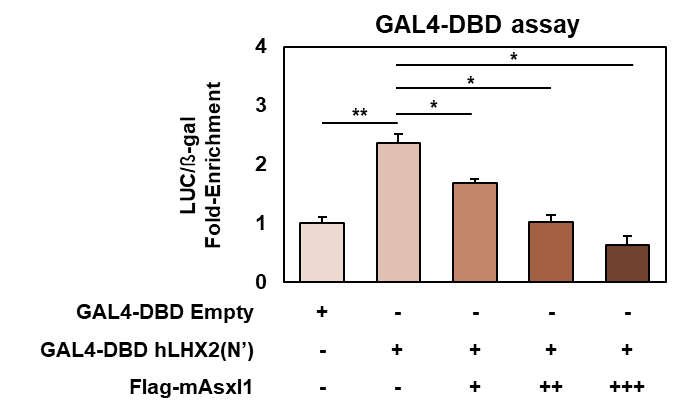


**Supplementary Figure 5. Effect of Asxl1 on the transcriptional activity of GAL4-hLHX2.** Gal4 DBD-fused hLHX2 **aa 1–174** were transfected into **293T cells together with increasing amounts of Flag-mAsxl1 expression vector**, and **GAL4 activity** was measured by luciferase assay and normalized using a **β-galactosidase** assay. Data were calculated as described in Material and method (n=3, *p < 0.05, **p < 0.01)


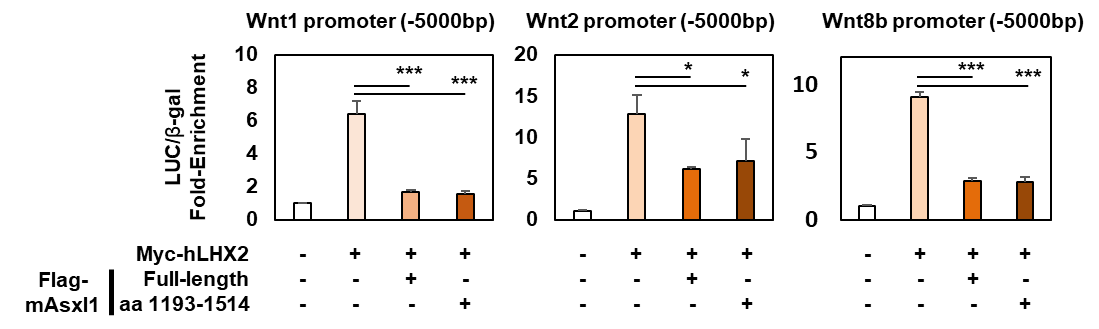


**Supplementary Figure 6. Transcriptional repression by the Asxl1 aa 1193–1514 fragment.** Luciferase reporter assays were performed using Wnt1, Wnt2, and Wnt8b regulatory regions in the presence of Myc-hLHX2, Flag-tagged full-length Asxl1, or the C-terminal Asxl1 fragment (aa 1193–1514). The Asxl1 fragment exhibited comparable transcriptional repression to the full-length protein. Data are presented as mean ± SD (n = 3). *p < 0.05, ***p < 0.001.


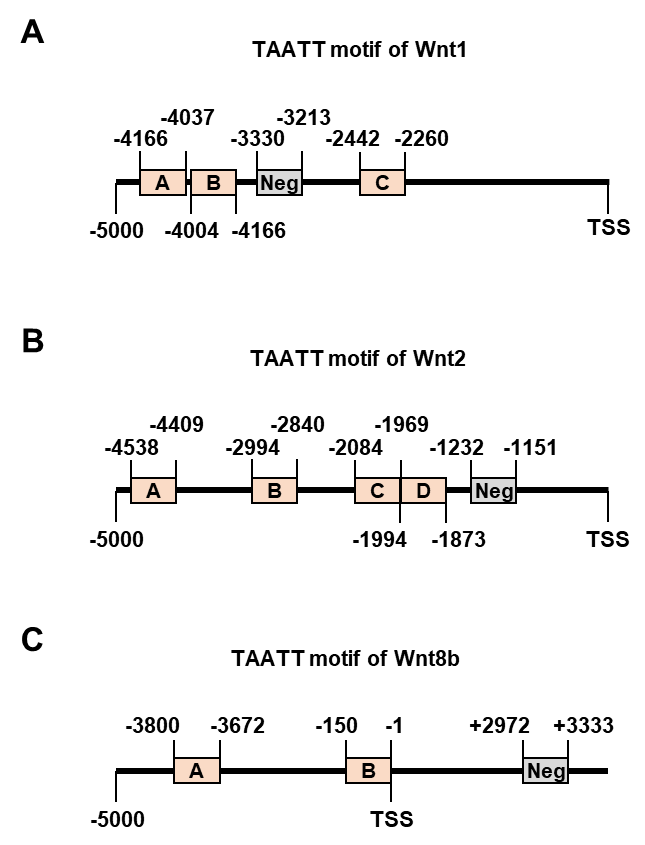


**Supplementary Figure 7. Schematic representation of primer designs including the Lhx2 binding motif.** (A–C) ChIP-PCR target regions including **Lhx2 binding motif TAATT** in the murine Wnt1 (A), Wnt2 (B) and Wnt8b (C) gene regulatory regions.
